# Task-Aware Low-Light Image Enhancement Method for Underground Coal Mine Monitoring

**DOI:** 10.3390/s26061886

**Published:** 2026-03-17

**Authors:** Zhirui Yan, Yaru Li, Hongwei Wang, Zhixin Jin, Lei Tao, Yide Geng

**Affiliations:** 1 College of Mechanical Engineering, Taiyuan University of Technology, Taiyuan 030024, China; 2 Center of Shanxi Engineering Research for Coal Mine Intelligent Equipment, Taiyuan University of Technology, Taiyuan 030024, China; jinzhixin@tyut.edu.cn (Z.J.); taoted@163.com (L.T.); gengyide@tyut.edu.cn (Y.G.); 3 College of Safety and Emergency Management and Engineering, Taiyuan University of Technology, Taiyuan 030024, China; 18334523129@163.com; 4 Xinjiang Intelligent Equipment Research Institute, Aksu 843000, China; 5 State Key Laboratory of Intelligent Mining Equipment Technology, Taiyuan 030032, China

**Keywords:** low-light image enhancement, Zero-DCE model, unsupervised learning, object detection, task awareness

## Abstract

Video AI recognition is crucial for coal mine safety, but complex environments often yield low-quality images, hindering intelligent monitoring. Existing enhancement methods typically focus on image quality alone, lacking adaptability to specific tasks. Therefore, we propose Mine-DCE-YDT: a task-aware low-light image enhancement model that jointly optimizes enhancement with downstream object detection, ensuring enhanced images are both visually clearer and more conducive to accurate detection. Firstly, an improved Zero-DCE algorithm (Mine-DCE) is presented by introducing a Brightness-aware Mask Coordinate Attention (BMCA) module to improve illumination balance in the Value channel of the HSV image and a Multi-scale Detail Enhancement (MDE) module to reinforce textures and suppress noise. Then, Mine-DCE is co-modeled with YOLOv11n by training end-to-end via a joint loss fusing detection and enhancement quality losses to form Mine-DCE-YDT, which can enhance specific details containing image detection targets. Experimental results show that compared with Zero-DCE, Mine-DCE-YDT achieves reductions of 9.5% in NIQE and 35.5% in BRISQUE on the custom-constructed MineDataset and exhibits great enhancement performance on the public dataset LOL-V1. For the miner detection task in MineDataset, the integration of Mine-DCE-YDT with YOLOv11n achieves increases of 2.8% and 8.3% in mAP@0.5 and mAP@0.5:0.95, demonstrating its effectiveness in enhancing task-critical image features.

## 1. Introduction

Coal serves as China’s primary energy source, making coal mine safety production pivotal for the energy sector’s stability [1]. Consequently, safety management stands as a crucial concern within the coal mining industry. With the development of intelligent coal mines, AI analysis and recognition technology for monitoring videos has become an important measure to ensure coal mine safety production [2]. However, underground environments are often affected by complex disturbances, such as insufficient illumination, suspended dust, and water mist dispersion, leading to pronounced degradations in surveillance images, including nonuniform illumination, color distortion, amplified noise, and loss of detail. These issues seriously interfere with the effectiveness of subsequent video AI recognition tasks, such as object detection and semantic segmentation, making it difficult to ensure real-time monitoring and effective warning of coal mine safety production [3]. Image enhancement technology serves as a crucial pre-processing step for improving overall image quality. Therefore, researching low-light image enhancement methods suitable for mining environments to improve image quality and AI recognition task availability is a key challenge that urgently needs to be overcome in the field of intelligent video surveillance in coal mines [4].

Existing image enhancement algorithms for coal mines mainly face the following problems: (1) Heavy reliance on paired images. Algorithms typically rely on high-quality reference images to improve enhancement performance, but it is difficult to obtain paired reference images in real coal mining scenes. (2) Difficulty addressing complex scenes. The environment of the coal mine working face has nonuniform illumination, large water mist and high dust concentrations; these factors will seriously affect the effectiveness of these image enhancement algorithms. (3) Isolation from downstream high-level vision tasks [5]. Image enhancement means to improve the visual quality of an image, but it also means to make the image suitable for further processing. Currently, most studies focus solely on low-light image enhancement in coal mines and neglect coordinated optimization with core visual analysis tasks. Although it can improve image appearance, excessive smoothing of structural textures, introduction of artifacts, or distortion of key target shapes can degrade the performance of downstream recognition algorithms [6]. This isolation highlights the urgent need for a task-aware enhancement paradigm that takes downstream detection requirements as guidance, rather than optimizing only for visual quality.

Although recent large foundation vision models, such as the Segment Anything Model (SAM) [7] and DINOv2 [8], have demonstrated exceptional performance across various tasks, their substantial computational overhead and memory footprint pose significant challenges for deployment in resource-constrained and real-time industrial scenarios. In underground coal mine monitoring, where hardware resources are limited and low-latency inference is critical for timely safety warnings, lightweight models like YOLOv11n are often the more practical and preferred choice. Therefore, our work focuses on optimizing a lightweight enhancement-detection pipeline compatible with such efficient detectors.

To address the above challenges, this paper proposes a task-aware low-light image enhancement method for underground coal mine monitoring. Rather than optimizing solely for perceived image quality, the method incorporates feedback from detection tasks and builds a collaborative optimization framework linking enhancement and detection, which can effectively improve object detection performance while enhancing image quality. The main contributions of this paper are summarized as follows:

(1) A new, improved low-light image enhancement module, Mine-DCE, based on Zero-Reference Deep Curve Estimation (Zero-DCE), is proposed. To reduce color distortion, Mine-DCE processes images in the HSV color space. It applies a Brightness Mask-guided Coordinate Attention (BMCA) module to the Value channel (V) for adaptive illumination balancing and incorporates a Multi-scale Detail Enhancement (MDE) module to preserve textures while suppressing noise. The proposed method can effectively improve the image quality in the coal mine underground.

(2) A task-aware low-light image enhancement algorithm, Mine-DCE-YDT, for underground coal mines is introduced. This algorithm achieves joint training optimization of the image enhancement model Mine-DCE and the object detection model YOLOv11n by constructing a joint loss function that integrates detection loss and enhancement quality loss. The model can selectively enhance only those features that lead to improved image detection by end-to-end dynamic learning.

(3) The image enhancement performance of the proposed model was validated on a custom-constructed MineDataset and the publicly available low-light image dataset LOL-V1, and the improvement in the target detection algorithm performance by the proposed strategy was verified on the MineDataset for underground personnel detection tasks in coal mines. Experiments using our proposed enhancement Mine-DCE-YDT show great results on all the datasets and effectively improve the accuracy of object detection algorithms.

The remainder of this paper is organized as follows. Section 2 overviews related work. Section 3 details our proposed enhancement method. Experiment and results analysis are presented in Section 4. Finally, Section 5 concludes this paper.

## 2. Related Work

### 2.1. Low-Light Image Enhancement

The issue of image degradation under low light conditions has been extensively addressed through techniques such as histogram equalization [9], gamma correction [10], Retinex theory [11], wavelet transform [12], and multi-exposure fusion [13]. These methods typically depend on fixed rules or manual priors. While they can improve overall brightness, they often result in detail loss, color distortion, and noise amplification. Furthermore, their generalization and stability are constrained in the complex lighting and high-noise environments characteristic of underground coal mines.

To overcome traditional limitations, deep learning enhancement methods have gained prominence. Among supervised learning approaches, LLNet [14] necessitates paired samples, which are often challenging to obtain in practice, and URetinex-Net [15] is capable of learning Retinex decomposition to enhance details, but its illumination estimation is sensitive to noise, leading to uneven enhancement in high-noise environments. Moving to unsupervised learning methods, EnlightenGAN [16] employs adversarial training for illumination restoration; however, this approach results in a complex and resource-intensive model. For self-supervised learning methods based on physical priors, IceNet [17] iteratively optimizes contrast, yet it relies on manual interaction and lacks automated adaptability. Finally, in the zero-reference learning paradigm, Zero-DCE [18] offers lightweight enhancement but struggles with insufficient contrast and detail recovery in extremely low-light coal mine environments, while SCI [19], which focuses on self-calibrating illumination estimation, enhances consistency but is susceptible to local overbrightness and detail loss in extremely low-light conditions.

### 2.2. Low-Light Image Enhancement Method in Underground Coal Mines

The underground environment of coal mines is characterized by extremely uneven lighting and severe interference from dust and water mist, which leads to widespread phenomena such as insufficient contrast, detail degradation, and noise amplification in monitoring images, seriously restricting the subsequent implementation of safety monitoring tasks. In response to this special scenario, relevant research in recent years has focused on improving the visual quality of low-light images to meet the requirements of underground safety monitoring.

Yang et al. [20] proposed a zero-reference learning image enhancement method for low-light personnel monitoring in underground coal mines, combining local pixel-level enhancement with a global adjustment mechanism based on Transformer to alleviate uneven enhancement and color distortion in bright and dark areas and reduce the impact on subsequent target detection. Tian [21] et al. proposed a target detection method for low-light underground mine environments. By introducing the zero-reference learning image enhancement network LMIENet to adaptively enhance the brightness and details of low-light images, and combining it with a lightweight target detection model, they improved the detection accuracy and real-time performance in complex mine environments. Zhao [22] et al. proposed a CycleGAN low-light image enhancement method for foreign object detection in underground coal mine belt conveyors. By integrating unsupervised learning with multi-scale feature enhancement, global luminance adjustment, and self-attention mechanisms, it aims to improve image quality under complex lighting conditions and enhance the performance of subsequent target detection.

### 2.3. Joint Multi-Task Learning

The core of multi-task learning is to jointly handle multiple related tasks through shared feature representations and to enhance learning efficiency and prediction accuracy by leveraging complementary information among tasks. It has been widely applied in computer vision dense prediction tasks such as classification, segmentation, and detection.

In response to the collaborative requirements of low-illumination enhancement and downstream tasks, a series of advanced multi-task joint optimization methods have emerged in recent years. Liang [23] et al. proposed the SCL-LLE framework, which uses contrastive learning, semantic luminance consistency, and feature preservation as multi-task joint constraints. It optimizes low-illumination enhancement by using unpaired positive and negative samples and combines semantic segmentation networks to ensure uniform luminance in similar regions, achieving a synergistic improvement in enhancement quality and downstream semantic segmentation. Trinh [24] et al. proposed an object tracking framework that integrates the real-time tracking algorithm with a dark channel prior image dehazing module, which effectively removes haze caused by adverse weather conditions, enhancing the performance of the object tracking model.

Although existing multi-task joint learning methods have achieved enhanced collaboration with downstream tasks in general low-light scenarios, they have not fully adapted to the unique scene characteristics of extreme light, high noise and strong interference in underground coal mines.

To clearly highlight the research gaps addressed in this work, a comprehensive summary of representative related methods is presented in Table 1.

## 3. Methodology

### 3.1. Overall Framework

**Figure 1:**
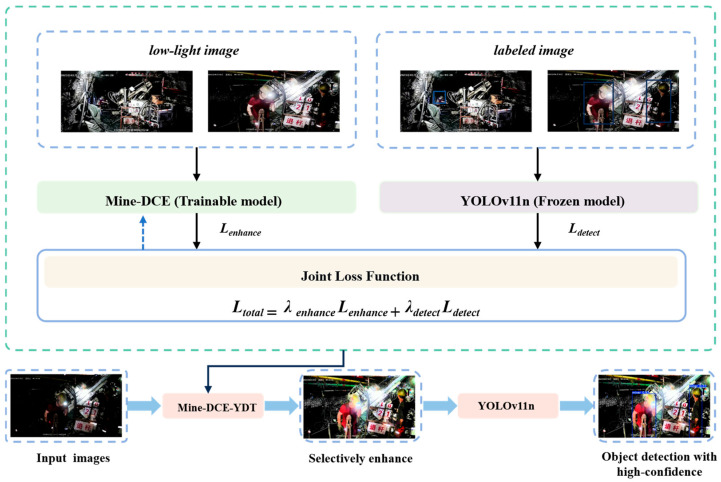
Mine-DCE-YDT: Joint optimization framework for enhancement and detection.

Aiming at the problems that traditional image enhancement methods in low-illumination scenes of underground coal mines are disconnected from target detection tasks, and that the enhancement results struggle to balance visual quality and detection performance, this paper proposes Mine-DCE-YDT, a detection-aware joint optimization model for low-light image enhancement and target detection, as illustrated in Figure 1. The model features a dual-flow design: an end-to-end inference pipeline for generating final detection results, and a closed-loop optimization module (the joint loss mechanism) for training the enhancement network. It takes semantic feedback from downstream detection tasks as the core constraint, guiding the enhancement network to generate detection-targeted, selectively enhanced image representations that better align with the feature extraction preferences of the detector while improving image visibility, ultimately achieving collaborative optimization of enhancement and detection performance.

As shown in the figure, the framework consists of three tightly coupled components: Mine-DCE (trainable model), YOLOv11n (frozen model), and the detection feedback-guided joint optimization mechanism.

As the only trainable component of Mine-DCE-YDT, Mine-DCE is built on an improved Zero-DCE architecture. It takes low-light underground images as input, and through targeted structural improvements (adapted to the underground scene characteristics of uneven illumination and strong noise), it generates enhanced images that stably correct brightness, retain detailed information, enhance personnel target features, and suppress noise interference, laying a high-quality foundation for subsequent detection tasks. The enhancement process is supervised by an enhancement loss $L_{enhance}$, which guides the module to learn robust enhancement capabilities.

YOLOv11n plays dual roles in the model. In the end-to-end inference pipeline, it receives selectively enhanced images from Mine-DCE-YDT and performs forward inference to output object detection results with high confidence. In the closed-loop optimization flow, it takes both enhanced images and labeled ground truth images as input, calculates detection loss $\;L_{detect}$ by comparing predictions with ground truth, and provides this loss as a feedback signal to Mine-DCE-YDT’s joint loss mechanism. The detector parameters remain frozen throughout to ensure the stability of detection feature distribution and avoid detector drift caused by small-scale underground datasets.

Detection feedback-guided joint optimization mechanism (internal to Mine-DCE-YDT): As the core optimization module of Mine-DCE-YDT, it constructs a joint loss function to apply both enhancement quality constraints and detection task feedback to the Mine-DCE enhancement network. The joint optimization objective is defined as:

(1) $$L_{total}=\lambda_{detect}L_{detect}+\lambda_{enhance}L_{enhance}$$

where $L_{detect}$ denotes the detection loss from YOLOv11n, $L_{enhance}$ denotes the enhancement quality loss from Mine-DCE, and $\lambda_{enhance}$ and $\lambda_{detect}$ are weighting coefficients. This mechanism propagates detection loss feedback to the enhancement network, enabling Mine-DCE to learn features that are more favorable for downstream detection.

### 3.2. Improved Zero-DCE Network Structure

Zero-DCE is a lightweight, no-reference low-illumination enhancement algorithm. It estimates a set of differentiable luminance adjustment curves through a deep network, performs pixel-by-pixel luminance correction on the input image, and enhances the naturalness, exposure consistency and color stability of the result with the constraint of no-reference loss. This work retains the overall enhancement paradigm and no-reference loss framework of Zero-DCE and only improves its network structure to enhance adaptability to underground coal mine scenarios.

**Figure 2:**
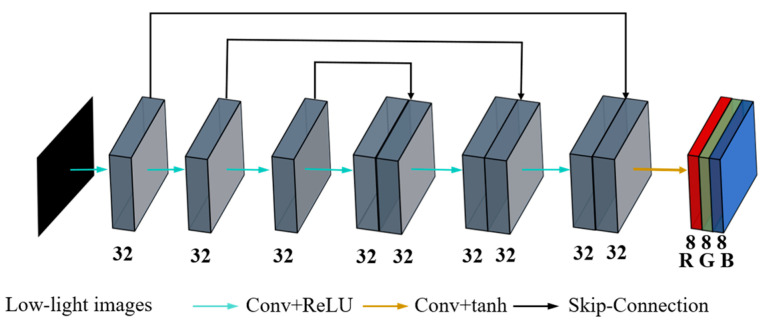
Zero-DCE model structure.

The original Zero-DCE is shown in Figure 2. DCE-Net adopts a symmetrical structure, including seven layers of convolutional modules. The first six convolutional layers are composed of 32 3 × 3 convolutional kernels with a step size of 1, and the ReLU activation function is used to activate between the convolutional layers. The 7th convolutional layer consists of 24 3 × 3 convolutional kernels with a step size of 1 and is activated using the tanh activation function. The first six layers of the network use hop connections to concatenate the outputs of the third and fourth layers, the second and fifth layers, and the first and sixth layers together, which are respectively used as the inputs of the fifth to seventh layers. The 7th layer outputs three color channels: R, G, and B (red, green, and blue, respectively).

**Figure 3:**
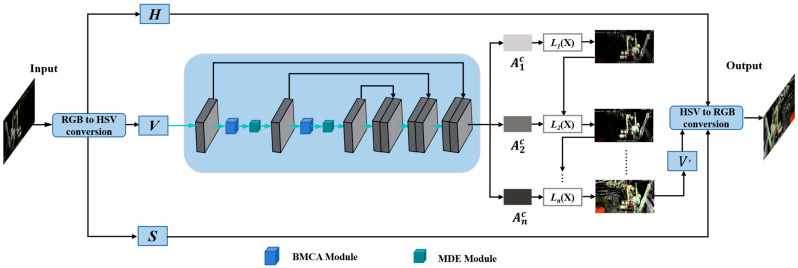
Mine-DCE: Process of improved Zero-DCE for mine low-light image enhancement.

The monitoring images in underground coal mines usually have the characteristics of coexisting bright and dark areas, significant local over-exposure/under-exposure, and strong coupling of noise and details. Directly using the original Zero-DCE is prone to problems such as color cast, uneven brightness correction, and weakened details. To this end, we make structural improvements from three aspects based on Zero-DCE, as illustrated in Figure 3: We introduce the HSV Value channel decoupling strategy in the enhancement process, enabling the enhancement process to maintain a stable color distribution while increasing brightness, and suppressing the common color drift phenomenon in underground low-illumination enhancement from the source. In the shallow/middle layer of the network (in the second and third layers of Zero-DCE), the BMCA module is introduced, enabling the network to adaptively enhance and suppress the dark and bright areas based on the luminance distribution, thereby improving the spatial consistency of luminance correction. Immediately after, the MDE module is added to enhance texture and structure expression through multi-scale feature extraction and fusion and to reduce the noise amplification effect in low-light enhancement.

### 3.3. Brightness Mask-Guided Coordinate Attention Module (BMCA)

The monitoring images in underground coal mines generally have a significant uneven lighting problem of “under-exposure in dark areas and over-exposure in bright areas”. This problem not only affects the overall visibility of the image, but it also leads to insufficient restoration of details in dark areas and excessive enhancement in bright areas during the low-light enhancement process [25], thereby weakening the feature discrimination ability of the subsequent object detection model. Therefore, identifying how to explicitly perceive and regulate the luminance distribution at the feature level is the key issue in achieving balanced illumination enhancement.

Traditional coordinate attention (CA) [26] can effectively capture the long-range dependency of features by encoding spatial position information in the horizontal and vertical directions, achieving good results in various visual tasks. However, the generation of its attention weights is completely dependent on the feature response itself, lacking an explicit modeling mechanism for the physical illumination distribution. In the underground coal mine scene, high-reflective areas, such as the direct illumination of miner’s lamps, reflective vests, and metal equipment, often generate strong local responses, making the attention mechanism prone to overfocus on these “ineffective high-brightness areas” [27] while allocating insufficient attention resources to the details of the truly needed dark areas, thereby limiting the light balance ability of low-illumination enhancement.

For this purpose, based on CA, this paper proposes the coordinate attention mechanism of BMCA. This module introduces a parallel luminance perception branch, embedding the data-driven illumination statistical prior [28] in a differentiable manner into the attention weight generation process. This enables the attention distribution to not only be influenced by feature semantics but also have the explicit perception ability of the luminance distribution, thereby achieving the expansion from “position perception” to “illumination-position collaborative perception”.

The BMCA module is integrated into the middle structure of the improved Zero-DCE network (after the second and third convolutions). The features at this stage have initially integrated spatial context information and contain relatively complete contour and texture details, but they have not yet been abstractly represented as high-level semantic representations. Introducing luminance perception modulation at this stage can guide the network to focus on dark area information [29] and suppress overresponse in bright areas from the source while retaining the detailed structure. It is an ideal position for enhancing illumination balance.

Before the BMCA module design, this paper carried out key pre-processing on the basic enhancement process: converting the input image from the RGB color space to the HSV color space to achieve decoupling of brightness and color information. The Value channel (*V*) in the HSV space directly corresponds to the physical light intensity, while the hue (*H*) and saturation (*S*) mainly describe the intrinsic color attributes. This decoupling strategy not only alleviates the color deviation problem caused by the coupling of the RGB three-channel from the source, but it also provides an input basis directly corresponding to the physical light intensity for the subsequent luminance perception branch.

The abovementioned color space transformation and brightness enhancement process can be expressed as:

(2) $$\begin{array}{c} I_{HSV}=T_{RGB\to HSV}\left(I_{RGB}\right)\\ V^{\prime }=\Phi (V;\theta )\\ I_{RGB}^{\prime }=T_{HSV\to RGB}\left(\left[H,S,V^{\prime }\right]\right) \end{array}$$

where $T_{RGB\to HSV}\left(\cdot \right)$ and $T_{HSV\to RGB}\left(\cdot \right)$ respectively represent the forward transformation and inverse transformation operations of the color space. $\Phi \left(V;\theta \right)$ represents the nonlinear luminance enhancement function parameterized by the improved deep network parameter θ. This function takes the decoupled luminance channel V as input and, by integrating modules such as BMCA, outputs an adaptive and illumination balanced enhancement result $V^{\prime }$.

**Figure 4:**
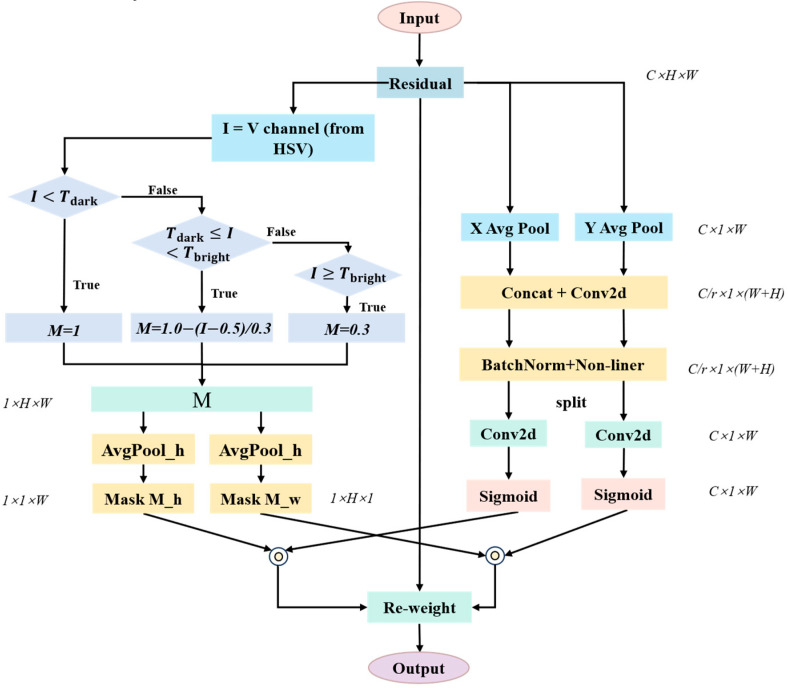
BMCA module structure.

The overall structure of the BMCA module is shown in Figure 4. It adopts a parallel dual-branch design, including a coordinate attention branch and a luminance mask branch. The former inherits the core idea of CA and is used to model the spatial dependency relationship of features. The latter is responsible for parsing the luminance information from the features and generating the luminance mask for modulating the attention weights. The outputs of the two branches are fused in the modulation stage, enabling the final attention weights to possess both semantic relevance and light perception capabilities simultaneously.

Given the input feature $F\in R^{C\times H\times W}$, the coordinate attention branch first performs global average pooling in both the horizontal and vertical directions to capture direction-aware long-range context information:

(3) $$F_{h}\;=\;\mathrm{AvgPoo}l_{h}\left(F\right),\;F_{w}\;=\;\mathrm{AvgPoo}l_{w}\left(F\right)$$

Subsequently, $F_{h}$ and $F_{w}$ are concatenated, and dimensionality reduction and feature fusion are performed through a shared 1 × 1 convolutional layer, with the output dimension being $R^{C/r\times 1\times (H+W)}$. After batch normalization (BN) and processing with nonlinear activation functions (such as Swish), this feature is split into two independent tensors and restored to the original number of channels *C* through two 1 × 1 convolutional layers. Finally, it is normalized by the Sigmoid function to generate the initial coordinate attention weight:

(4) $$A_{h}\in R^{C\;\times \;1\times \;W},\;A_{w}\in R^{C\;\times \;H\;\times 1}$$

Meanwhile, the luminance mask branch parses the luminance information I from the features (corresponding to the Value channel (*V*) in the HSV space), and based on the luminance histogram statistics of the mine dataset, constructs a differentiable piecewise linear mask function $M\left(I\right)$:

(5) $$M\left(I\right)=f\left(x\right)=\left\{\begin{array}{cc} 1.0, & I<T_{dark}\\ 1.0-\frac{\left(I-T_{dark}\right)}{{T_{bright}-T}_{dark}}, & T_{dark}\leq I<T_{bright}\\ k, & I\geq T_{bright} \end{array}\right.$$

where the thresholds $T_{dark}=0.5$ and $T_{bright}=0.8$ are the boundaries of the dark and bright areas determined based on the statistical observation of the dataset, and k=0.3 is the minimum retention coefficient of the bright area, which is used to ensure the continuity of the gradient flow. The design of this function is inspired by the JND (Just-Noticeable Difference) study [30]. It is worth noting that this suppression mechanism applies uniformly to all high-luminance regions, including specular reflections from equipment, thereby addressing potential glare issues without additional complexity.

The generated mask $M\left(I\right)$ is decomposed into $M_{h}$ and $M_{w}$ to match the dimensions of the coordinate attention weights. The outputs of the two branches are fused in the modulation stage, and the luminance mask recalibrates the initial coordinate attention weights for light perception:

(6) $$\tilde{A_{h}}=A_{h}⊙M_{h},\tilde{A_{w}}=A_{w}⊙M_{w}$$

where ⊙ represents element-by-element multiplication. The modulated attention weights $\tilde{A_{h}}$ and $\tilde{A_{w}}$ ultimately act on the input feature map, outputting enhanced features with balanced illumination:

(7) $$F^{\prime }=F⊙\tilde{A_{h}}⊙\tilde{A_{w}}$$

### 3.4. Multi-Scale Detail Enhancement Module (MDE)

In the task of low-illumination image enhancement in underground coal mines, there is often a significant contradiction between detail restoration and noise suppression. On the one hand, the contours, edge structures and local textures of personnel under low illumination conditions are usually relatively weak and need to be strengthened through enhancement operations. On the other hand, random noise components caused by sensor noise, dust scattering and low signal-to-noise ratios are widespread in underground images. Excessive enhancement can easily synchronously amplify the noise response, thereby leading to structural distortion and even interfering with the discrimination process of subsequent target detection models [31]. Therefore, identifying how to effectively suppress noise amplification while enhancing real details is one of the key challenges in low-light image enhancement.

Traditional enhancement methods based on single-scale features usually rely on a fixed receptive field for local information modeling, making it difficult to simultaneously take into account structural features at different levels. Although a smaller receptive field is beneficial for capturing fine-grained textures, it is highly sensitive to noise. A larger receptive field is more suitable for modeling the overall structure, but it is easy to overlook local details. This scale-constrained feature representation method makes it difficult for single-scale enhancement methods to achieve a balance between detail enhancement and noise suppression in complex underground scenarios.

To this end, this paper proposes the MDE module. Through a parallel multi-scale feature extraction and adaptive fusion mechanism [32], it effectively enhances the details of the real structure while maintaining computational efficiency and suppressing random noise. The core idea of MDE is that real details usually have structural consistency at multiple scales, while random noise often manifests as isolated high-frequency responses and is only significant at small scales. Based on this observation, multi-scale consistency can serve as an important criterion for distinguishing real details from noise.

**Figure 5:**
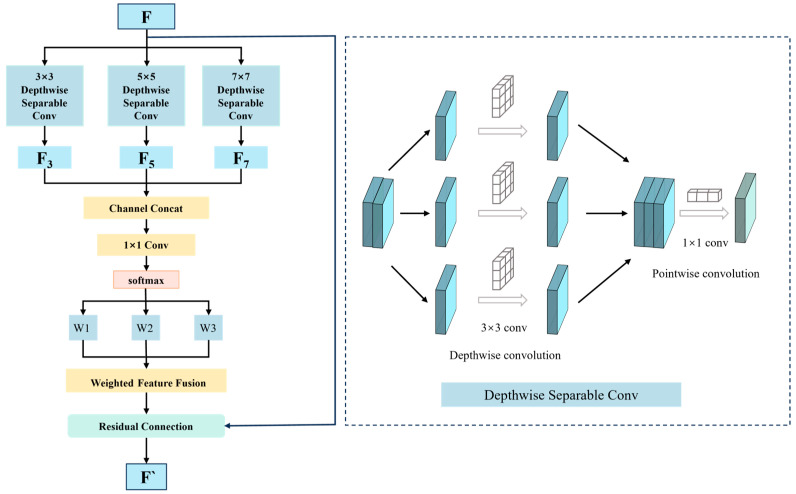
MDE module structure.

The structure of MDE is shown in Figure 5. The input feature map is as follows:

(8) $$F\in R^{C\times H\times W}$$

The module first feeds three parallel depth-separable convolutional [33] branches for processing. Each branch consists of two stages: Depthwise Convolution uses independent two-dimensional convolution kernels (with sizes of 3 × 3, 5 × 5, and 7 × 7 respectively) to extract spatial features from each input channel, focusing on capturing spatial details of different scales. Pointwise Convolution uses convolution to perform channel fusion on the output of depthwise convolution, integrating feature information between different channels.

Through the above operations, the three branches output features $F_{3}$, $F_{5}$ and $F_{7}$, corresponding to the feature information of the small, medium, and large receptive fields, which are respectively responsible for extracting fine textures, local structures, and global contours.

Subsequently, the output features of the three scales are concatenated along the channel dimension to form multi-scale fusion features:

(9) $$F_{\mathrm{concat}}=\mathrm{Concat}\left(F_{3},F_{5},F_{7}\right)$$

On this basis, the number of channels is compressed through the convolutional layer, and the spatial dimension is normalized by the Softmax function to generate pixel-level adaptive weight maps corresponding to the three scales $W_{1}$, $W_{2}$, and $W_{3}$:

(10) $$\left[W_{1},W_{2},W_{3}\right]=\mathrm{Softmax}\left({\mathrm{Conv}}_{1\times 1}\left(\mathrm{Concat}\left(F_{3},F_{5},F_{7}\right)\right)\right)$$

For any spatial position $\left(i,\;j\right)$, the weights satisfy the constraint conditions:

(11) $$W_{1}\left(i,\;j\right)\;+\;W_{2}\left(i,\;j\right)\;+\;W_{3}\left(i,\;j\right)\;=\;1$$

This adaptive mechanism enables the network to dynamically allocate the contribution degree of features at each scale according to the local characteristics of different spatial positions. The core of this mechanism lies in its noise discrimination ability: Real details usually exhibit structural consistency at multiple scales, and the network assigns balanced weights to them. Random noise often presents as isolated high-frequency responses and is usually only significant at the smallest scale ($F_{3}$). Through training, the network learns to recognize such patterns, automatically reduces the $W_{1}$ weights of the noise regions, and instead relies on features that are more consistent at medium and large scales, thus achieving a balance between detail enhancement and noise suppression.

Multi-scale feature weighted fusion: Each scale feature map $F_{3}$, $F_{5}$ and $F_{7}$ is weighted according to the corresponding weights $W_{1}\;,$ $W_{2},$ and $W_{3}$:

(12) $$\mathrm{fused}\text{\_}\mathrm{feat}\;=\;W_{1}\;⊙\;F_{3}\;+\;W_{2}\;⊙\;F_{5}\;+\;W_{3}\;⊙\;F_{7}$$

where ⊙ denotes element-wise multiplication (element-by-element multiplication). To prevent distortion in the fused features, residual connections are incorporated to perform weighted fusion of the fused multi-scale features and the original input features:

(13) $$F^{\prime }\;=\;F\;+\;\alpha \cdot \mathrm{fused}\text{\_}\mathrm{feat}$$

where α = 0.3 is the enhancement intensity coefficient, which is determined through grid search on the validation set. The enhancement intensity coefficient α was optimized via grid search on the validation set over [0.1, 0.5] with a 0.1 step size. Values below 0.2 caused insufficient detail enhancement, while α≥0.4 introduced noticeable noise artifacts. Among valid candidates, α = 0.3 was selected as optimal for its best balance of detail preservation and noise suppression. It not only retains the basic structure of the original features but also controls the extent of detail enhancement, and, at the same time, it improves the efficiency of gradient propagation, ensuring the stability of network training.

In this paper, the MDE module is embedded in the middle and shallow layers (after the second and third layers) of the improved Zero-DCE network. The features at this position contain rich low-level detail information, such as edges and textures. At this stage, multi-scale enhancement can provide a better feature basis for the subsequent estimation of luminance curves.

### 3.5. Joint Optimization Mechanism Guided by Detection Feedback

In underground coal mines, surveillance images often suffer from low illumination, dust, and water mist, leading to issues of poor contrast, lost details, and severe noise. This discrepancy causes traditional low-light enhancement results to misalign with the feature extraction requirements of downstream detectors [34], thereby hindering reliable target detection. To address this limitation, this paper proposes a detection-aware enhancement method that incorporates semantic feedback from a pre-trained YOLOv11n detector, employing an asymmetric joint optimization strategy consistent with the model in Figure 1: detector parameters remain frozen to provide stable error signals, while Mine-DCE is the only trainable module of the Mine-DCE-YDT model, updated iteratively via backpropagation of joint loss gradients.

The YOLOv11n detector is first independently fine-tuned for underground “miner” target detection. A warm-up training strategy is adopted to mitigate loss imbalance and premature feedback issues. For the first 10 epochs, only the enhancement loss $L_{enhance}$ is optimized to help Mine-DCE establish basic enhancement capabilities. Once the parameters stabilize, joint training commences, during which the YOLOv11n parameters remain frozen to prevent detector drift from small-scale underground datasets and ensure consistent feedback signals.

In each training iteration, a low-light image is fed into the Mine-DCE enhancement network to generate an enhanced image. The network adopts Zero-DCE’s no-reference enhancement loss as its basic constraint: $L_{enhance}$ comprises exposure control loss, spatial consistency loss, color constancy loss, and total variation smoothing loss, which collectively enforce rational brightness, structural continuity, and color stability to guarantee the visual quality of enhanced outputs. The enhanced image is then input to the frozen YOLOv11n detector for forward inference, producing detection predictions. The detection loss $L_{detect}$ is calculated against a labeled ground truth, consisting of bounding box regression loss and classification loss—this loss quantifies how effectively enhanced images support target recognition and localization from the detector’s perspective. Although the detector parameters are not updated, the forward pass constructs a complete computational graph, making the enhanced image’s detection loss differentiable; gradients are backpropagated to Mine-DCE via the chain rule, consistent with the training optimization flow of Mine-DCE-YDT.

The weighting coefficients in the joint loss function (Equation (1)), $\lambda_{enhance}=0.8$ and $\lambda_{detect}=0.3$, were determined through a grid search on the validation set. We evaluated $\lambda_{enhance}$ in the range of [0.5,1.2] and $\lambda_{detect}$ in [0.1,0.5], with the objective of balancing detection performance and visual quality. Our grid search revealed that $\lambda_{detect}\geq 0.4$ degraded perceptual image quality (higher NIQE), as the enhancement network over-emphasized detection-specific features at the expense of naturalness. Conversely, $\lambda_{detect}\leq 0.2$ provided insufficient task guidance, with mAP@0.5 gains of less than 1% vs. Mine-DCE alone. For $\lambda_{enhance}$, values below 0.7 caused insufficient enhancement (higher BRISQUE), while values above 1.0 brought marginal detection improvement but over-smoothed fine details. The combination of $\lambda_{enhance}=0.8$ and $\lambda_{detect}=0.3$ achieved the best balance between detection accuracy and visual quality. The selected asymmetric weighting reflects the design principle that the enhancement loss prioritizes establishing basic visual clarity, while the detection loss offers finer, task-aware guidance to refine the feature representation. Under this optimization mechanism, Mine-DCE gradually learns to generate image features that facilitate target discrimination. The experimental results confirm that this joint optimization strategy significantly improves the accuracy and robustness of underground low-light target detection, verifying the effectiveness of integrating detection feedback.

## 4. Experiment and Analysis

### 4.1. Experimental Settings

In terms of the configuration of the experimental environment, this study adopts a computing platform equipped with the Windows 10 operating system. The hardware core includes an Intel Xeon Silver 4210R@2.40GHz processor, an NVIDIA Tesla V100 PCIe graphics card, and 32 GB of system memory. The software level is based on the Windows 10 (version 10.0.19045) platform, uses the PyCharm 2021.3.3 development environment, takes Python 3.10.18 as the programming language, and relies on the PyTorch 2.4.1 deep learning framework and CUDA 12.4 computing architecture. GPU-accelerated training is achieved in conjunction with CUDA toolkit 11.7 and cuDNN 9.0.1. During the training phase, a well-verified parameter configuration was adopted: the initial learning rate was set to 0.0001, the number of training epochs was set to 100, the batch size was set to 8, and the Adam optimizer was selected. This combination effectively ensured the stability of the training process and promoted the rapid convergence of the model, providing an important guarantee for the reliability of the experimental results.

Two datasets were employed for validation in this experiment. For the public LOL-V1 dataset, only its officially divided 15 pairs of low-light/normal-light test images were utilized. The other dataset was constructed from the public DsDPM66 [35] dataset tailored for underground coal mine drilling scenarios. Since most images in DsDPM66 are captured under strong industrial lighting, they represent normal illumination conditions. To simulate the challenging low-light scenarios with nonuniform illumination and coal dust haze in underground coal mine areas without direct lighting, we screened 3750 “miner” images as the base MineDataset. Specifically, we further darkened these images via gamma transformation combined with local brightness differences to mimic the nonuniform low-light characteristics of underground coal mines and then added Gaussian noise with random intensity and low-intensity salt-and-pepper noise to the degraded images [36] to generate the coal mine low-light dataset. This luminance degradation process matching real underground low-light characteristics was applied to 1500 randomly selected samples (40% of the base set), forming a mixed normal/low-light dataset. This mixed dataset was split into training (2625), validation (750) and test (375) sets at a 7:2:1 ratio via stratified sampling, with consistent low-light image proportions across all subsets. The validation covers the constructed coal mine low-light test set and the public LOL-V1 low-light test set.

To verify the effectiveness of the proposed method, we selected several representative algorithms for quantitative and qualitative comparative experiments. The compared algorithms include two zero-reference learning-based methods (Zero-DCE and SCI), two supervised learning-based methods (URetinex-Net and Retinex-Net), one unsupervised GAN-based method (EnlightenGAN), and one self-supervised learning method (IceNet). These algorithms were evaluated for their image enhancement performance, and the rationality of the proposed network structure was further validated through ablation experiments.

To comprehensively evaluate the performance of each algorithm, this paper adopts five mainstream image quality assessment indicators, covering both no-reference and full-reference evaluation systems. The no-reference evaluation metrics include NIQE and BRISQUE, which are used to assess the naturalness and perceived quality of enhanced images. The lower the values of both, the closer the statistical characteristics of the image are to natural images, and the better the visual effect. The full-reference evaluation metrics include PSNR, SSIM and LPIPS, which quantify the differences between the enhanced image and the reference image from the perspectives of signal-to-noise ratio, structural similarity and visual perception, respectively. The higher the PSNR value and the closer the SSIM value is to 1, the lower the degree of image distortion and the better the detail fidelity. The smaller the LPIPS value, the closer the enhanced image is to the real image in terms of visual perception.

### 4.2. Ablation Experiment

**Figure 6:**
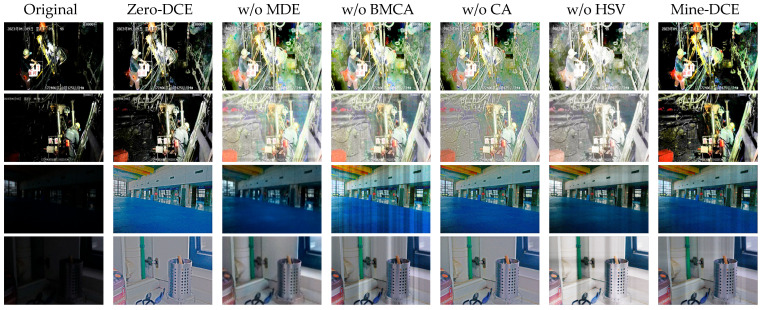
Visualization of the ablation experiment.

To assess the contribution of each module to image enhancement, we conducted ablation experiments in which the HSV-space perceptual enhancement module, the CA coordinate attention mechanism, the BMCA module and the MDE module were sequentially removed to evaluate their effects on image enhancement metrics. Table 2 presents the quantitative comparison results after the removal of each module, and a visualization of the ablation study is shown in Figure 6.

The quantitative ablation results (Table 2) provide definitive evidence for the function of each module. On the MineDataset, removing the HSV module causes NIQE to increase from 4.60 to 5.26, quantitatively proving that this module is crucial for maintaining the natural statistical properties of the image and preventing color casts. Removing the CA module worsens BRISQUE from 18.94 to 27.19, indicating that the spatial–directional information it provides is essential for maintaining illumination consistency and avoiding local distortions. Removing the BMCA module leads to the most severe BRISQUE degradation (to 32.19), highlighting the core role of its luminance-aware and cross-spatial attention mechanism in noise suppression and dynamic range balancing. After removing the MDE module, although NIQE remains acceptable, the significant decline in PSNR and SSIM on the LOL-V1 dataset demonstrates that this module is indispensable for restoring details aligned with the true structure. The complete model achieves optimal values across all key metrics, confirming the effectiveness of module synergy.

The visual effects after removing individual modules are shown in Figure 6. From the visual assessment, removing the MDE module leads to insufficient detail restoration, resulting in overly smoothed textures and impaired structural preservation; removing the BMCA module yields inadequate brightening of dark regions and overexposure in bright areas, markedly exacerbating local illumination inconsistencies, which intuitively demonstrates the critical roles of these two modules in detail recovery and illumination balancing. In addition, removing HSV color decoupling produces pronounced color shifts, and removing the CA module prevents adequate capture of spatially varying illumination, causing decreased texture uniformity and inaccurate localized brightening, further confirming the importance of each component for maintaining color fidelity and structural integrity.

### 4.3. Quantitative Analysis

To quantitatively analyze the effectiveness of the proposed algorithm, we conduct quantitative evaluations on the MineDataset and LOL-V1 datasets. The quantitative comparison results are shown in Table 3. Mine-DCE performs the best on the MineDataset, with NIQE and BRISQUE values of 4.6012 and 18.9365, respectively, both of which are optimal among all compared methods. Compared with the baseline Zero-DCE model, these two indicators are reduced by 8.2% and 35.8%, respectively. It also outperforms EnlightenGAN by 5.4% and 27.9% in the two metrics, demonstrating its excellent adaptability to coal mine scenarios. After introducing the detection loss, the Mine-DCE-YDT model further improves the NIQE index to 4.5374, while the BRISQUE value slightly increases to 19.0268. Compared with the original Zero-DCE model, Mine-DCE-YDT achieves a 9.5% reduction in NIQE and a 35.5% reduction in BRISQUE, with the elevated NIQE reduction reflecting the enhanced naturalness of images under the guidance of detection feedback and the slight fluctuation in BRISQUE being a reasonable trade-off for optimizing the representation of target features critical for downstream detection. This indicates that the introduction of the detection task enhances the representation of target structures without significantly affecting the overall naturalness of the image.

On the LOL-V1 dataset with reference images, URetinex-Net achieves the best performance on supervised evaluation metrics such as PSNR and SSIM. Among all compared methods, the LPIPS of Mine-DCE-YDT is 0.1589, which is approximately 27% lower than that of the baseline Zero-DCE (0.2174), demonstrating that the improved model still exhibits good cross-dataset generalization ability. Overall, although the improved model is not optimal in terms of supervised metrics, its robust performance on LOL-V1 validates its generalization ability on public datasets.

Notably, while Mine-DCE-YDT achieves state-of-the-art results on our custom MineDataset, its performance on the public LOL-V1 dataset, although robust, does not surpass specialized supervised methods like URetinex-Net. This is expected, as our model’s components—such as the BMCA luminance thresholds and MDE noise suppression—are specifically optimized for the challenging, nonuniform conditions of underground mines, which differ from the general low-light scenes in LOL-V1. This highlights the domain-specific nature of our approach.

### 4.4. Qualitative Analysis

**Figure 7:**
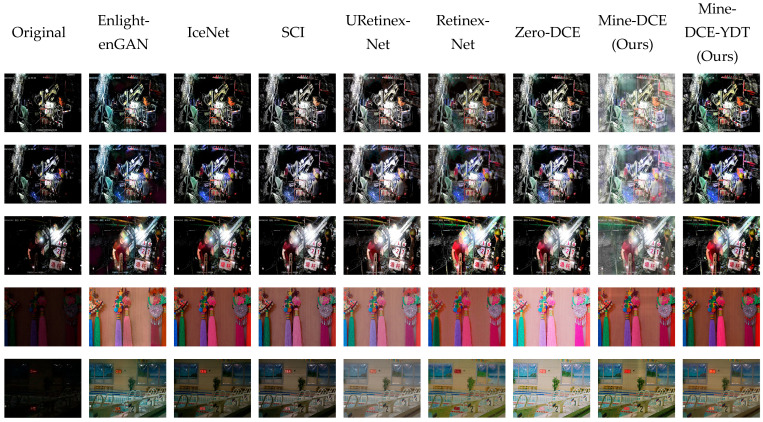
Visualization of the results produced by different low-light image enhancement algorithms on LOL-V1 and MineDataset.

Visual comparisons of different low-light enhancement algorithms on LOL-V1 and MineDataset are presented in Figure 7. While EnlightenGAN significantly improves overall brightness, its generative adversarial training often introduces pronounced edge blurring and unrealistic color shifts, likely due to the difficulty in constraining high-frequency details and color consistency in unpaired learning. IceNet achieves reasonable brightness and contrast adjustment; however, it tends to lose fine textures and cause localized color deviations, possibly because its iterative, interactive mechanism lacks sufficient constraints for detail preservation across diverse scenes. SCI balances global brightness and contrast effectively but fails to restore accurate colors, as its self-calibrated illumination estimation may not adequately model complex color interactions in low-light conditions. Retinex-Net enhances visibility but frequently introduces conspicuous color casts and blurring artifacts, stemming from its sensitivity to noise in the estimated illumination and reflectance components.

In contrast, our Mine-DCE produces visually more coherent results: it clearly restores details in dark regions while effectively suppressing overexposure in bright areas, yielding an overall tone closer to the true underground visual environment. This can be attributed to our HSV-based decoupling, which stabilizes color representation, and the BMCA module, which adaptively balances illumination. Furthermore, Mine-DCE-YDT sharpens edge contours and preserves subtle details in both dark and bright regions, benefiting from the detection-aware feedback that guides the enhancement toward maintaining structurally salient features. These visual improvements correlate directly with the observed gains in detection performance, confirming that our method not only enhances perceptual quality but also supports downstream analytical tasks.

### 4.5. Object Detection Experiment

**Figure 8:**
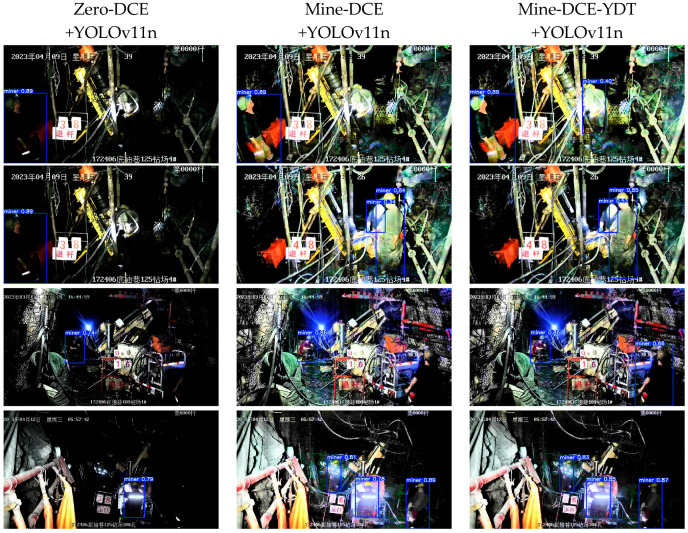
Comparison of recognition results of different algorithms.

To further evaluate the performance of the image enhancement method with detection task guidance in the underground personnel detection scenario of coal mines, YOLOv11n was used as the object detection model for miner detection in the MineDataset. The detection indicators are shown in Table 4, and the visual results are presented in Figure 8.

To thoroughly evaluate the contribution of enhancement to downstream detection, we compare the detection performance of the original low-light images and those processed by different enhancement strategies, using the same YOLOv11n detector. The results in Table 4 demonstrate that both Mine-DCE and Mine-DCE-YDT significantly improve all detection metrics compared to the original input, confirming that our enhancement strategy effectively mitigates low-light degradation and boosts detection robustness in underground coal mine scenarios.

Specifically, Mine-DCE increases mAP@0.5 from 0.948 to 0.962 (+1.5%) and mAP@0.5:0.95 from 0.795 to 0.839 (+5.5%), indicating better localization accuracy across varying IoU thresholds, while the rise in precision (0.977) and recall (0.932) further shows that enhanced images reduce false positives while recovering more true positives—critical for safety-critical underground coal mine monitoring, where missed or false detections could lead to safety hazards. In comparison, Mine-DCE-YDT, incorporating detection-aware feedback, achieves the best results: mAP@0.5 reaches 0.975 (+2.8% compared to the original input), mAP@0.5:0.95 rises to 0.861 (+8.3%), and precision and recall improve to 0.986 and 0.956, respectively. As shown in Figure 8, compared with Mine-DCE, Mine-DCE-YDT also yields higher detection confidence and fewer missed detections in visualizations. The notably larger gain in mAP@0.5:0.95 suggests that the model improves detection consistency under stricter localization criteria, reflecting enhanced structural integrity and boundary precision of target regions (e.g., miners and key equipment), which is attributed to the detection loss steering the enhancement network to preserve and sharpen features that are discriminative for detection—such as edges, textures, and contrast around miners—while suppressing irrelevant noise and artifacts.

Importantly, these detection gains align with the perceptual quality maintained in earlier quantitative results (e.g., competitive NIQE and LPIPS), illustrating that Mine-DCE-YDT successfully balances natural appearance, structural preservation, and detection-friendly representation. This makes it particularly suitable for vision-based underground coal mine monitoring systems, where both image interpretability (for human supervision) and machine analysis reliability (for intelligent detection) are required.

**Figure 9:**
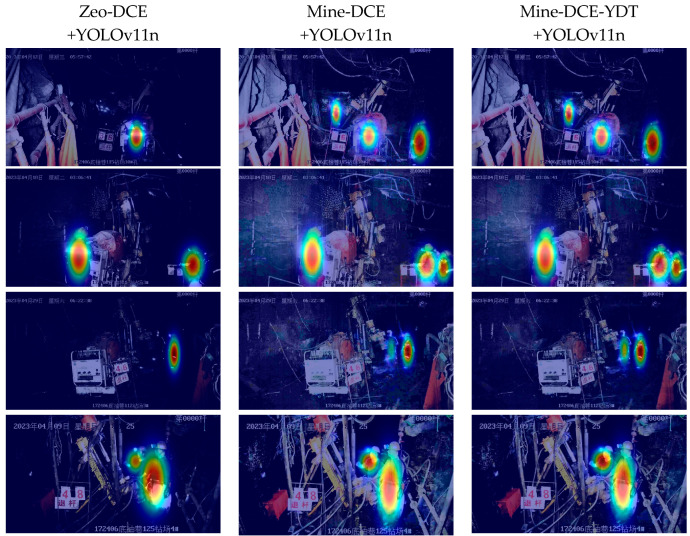
Visualization results of the Grad-CAM heat map.

To intuitively analyze the impact of the improved Mine-DCE-YDT module, we present the model’s decision-making process and its focus regions, and to further improve model interpretability, we adopt Grad-CAM heat maps for visualization. Figure 9 illustrates the Grad-CAM-based heat maps corresponding to three input conditions. For the original low-light image without enhancement, the detector’s activation regions are scattered and blurred with severe background noise, resulting in unfocused attention on the target area and degraded detection performance. After Mine-DCE enhancement, the target activation regions are moderately concentrated, and the image brightness is improved, yet background interference still exists, and the model’s attention is not fully locked on the target, leading to limited gains in detection accuracy. In contrast, Mine-DCE-YDT makes the detector’s activation regions highly concentrated, with background interference nearly eliminated, thus significantly boosting object detection performance.

### 4.6. Computational Complexity Analysis

To quantitatively assess the computational cost introduced by the BMCA and MDE modules, we compared the GFLOPs, parameters, and inference speed of the baseline Zero-DCE and our proposed Mine-DCE. All measurements were performed on an NVIDIA Tesla V100 GPU under the same experimental settings as the rest of our study, and the results were averaged over 100 inference runs.

As shown in Table 5, compared to the baseline Zero-DCE, our proposed Mine-DCE introduces a modest increase in computational cost, with GFLOPs increasing by 8.5% and parameters by 6.1%. This minimal overhead is a highly favorable trade-off for the significant gains in image quality (8.2% reduction in NIQE, 35.8% reduction in BRISQUE) achieved by the BMCA and MDE modules, as reported in Section 4.3.

Importantly, Mine-DCE achieves 307.69 FPS, which is 10.2 times higher than the real-time requirement of 30 FPS for video surveillance applications. This confirms that our proposed enhancement network, despite the added BMCA and MDE modules, remains exceptionally lightweight and well-suited for practical deployment in underground coal mine monitoring systems.

## 5. Conclusions

To address image degradation caused by low illumination, dust, and water mist in underground coal mine monitoring that impairs vision-based detection, this paper proposes Mine-DCE-YDT, a task-aware low-light enhancement model guided by detection-task feedback with an asymmetric joint optimization strategy. Built on the improved Zero-DCE (Mine-DCE), the model integrates the BMCA module for HSV Value channel (V) illumination balance and the MDE module for texture enhancement and noise suppression. It is co-modeled end-to-end with YOLOv11n via a joint loss fusing detection and enhancement quality losses, addressing the limitation of existing methods that lack adaptability to specific coal mine scenarios.

Experimental results show that compared with the original Zero-DCE model, Mine-DCE-YDT reduces NIQE by 9.5% and BRISQUE by 35.5% on the custom-constructed MineDataset and exhibits excellent enhancement performance on the public low-light image dataset LOL-V1. For underground coal mine personnel detection, integrating Mine-DCE-YDT into YOLOv11n improves mAP@0.5 and mAP@0.5:0.95 by 2.8% and 8.3%, respectively, which is attributed to its effective enhancement of image target features and focus on detection-critical details. The model effectively balances image perceptual quality and downstream object detection accuracy, providing reliable technical support for coal mine safety monitoring and intelligent development; its task-aware design can serve as a reference for other low-light industrial vision systems.

Despite its promising performance, this study has several limitations, including the frozen detector’s inability to adapt to new object classes, site-specific luminance thresholds, and unexamined temporal flickering in video streams. While the BMCA and MDE modules introduce additional computational overhead (+8.5% GFLOPs), the proposed Mine-DCE still achieves 307.69 FPS, which comfortably exceeds the real-time requirement of 30 FPS for video surveillance applications, confirming its suitability for practical deployment. Future work will address the aforementioned limitations through adaptive threshold mechanisms, video-level optimization, and extension to other downstream tasks.

## Figures and Tables

**Table 1:** Summary of related work and identification of research gaps.

	Representative Methods	Strengths	Weaknesses and Research Gaps
General Low-Light Enhancement	Histogram Equalization [9], Gamma Correction [10], Retinex [11], Wavelet Transform [12], Multi-exposure Fusion [13]	Improve global brightness and contrast; computationally efficient.	Often fail in extreme, nonuniform mine lighting; cause color distortion and noise amplification; disconnected from downstream tasks; unable to handle dust/water mist interference in mines.
Deep Learning-Based Enhancement	LLNet [14] (supervised), URetinex-Net [15] (supervised), EnlightenGAN [16] (unsupervised), IceNet [17] (self-supervised), Zero-DCE [18] (zero-reference), SCI [19] (zero-reference)	Superior enhancement quality; ability to learn complex mappings.	Supervised methods require paired data (difficult to obtain in mines); unsupervised/zero-reference methods struggle with extreme low-light conditions, local overbrightness, and detail loss in coal mine environments; still lack task awareness.
Mine-Specific Enhancement	Yang et al. [20] (Transformer-based), LMIENet [21] (Zero-DCE + detection), Zhao et al. [22] (CycleGAN-based)	Tailored for underground mine environments; improve visual quality for monitoring.	Primarily focus on perceptual quality; lack explicit, end-to-end joint optimization with specific high-level tasks like detection; some still rely on post-processing pipelines.
Joint Multi-Task Learning	SCL-LLE [23] (enhancement + segmentation), Trinh et al. [24] (dehazing + tracking)	Link enhancement with downstream tasks; leverage complementary information.	Designed for general low-light or specific tasks (segmentation/tracking); not fully adapted to the unique challenges of underground coal mines (extreme light variation, high noise, dust/water mist interference).
Ours (Mine-DCE-YDT)	BMCA + MDE + YOLOv11n joint optimization	Task-aware joint optimization with detection feedback; BMCA for illumination balance; MDE for detail enhancement and noise suppression; end-to-end training with detection loss; demonstrated gains in both image quality and detection accuracy.	

**Table 2:** Comparison of evaluation metrics for ablation experiments of each module. (The best indicators are displayed in bold. ↑ indicates that a higher value represents better performance; ↓ indicates that a lower value represents better performance).

Model	MineDataset		LOL-V1		
	NIQE ↓	BRISQUE ↓	PSNR ↑	SSIM ↑	LPIPS ↓
Original	5.3786	30.1968	7.8160	0.1948	0.5132
Zero-DCE	5.0139	29.5111	15.6161	0.7364	0.2174
*w*/*o* HSV	5.2606	25.6187	14.9356	0.7019	0.2175
*w*/*o* CA	4.9889	27.1863	17.3150	0.7603	0.1662
*w*/*o* BMCA	4.9965	32.1880	16.9841	0.6443	0.2332
*w*/*o* MDE	4.7618	23.3323	15.5214	0.6461	0.2396
Mine-DCE	**4.6012**	**18.9365**	**17.3150**	**0.7694**	**0.1584**

**Table 3:** Quantitative comparison of LOL-V1 and MineDataset. (The best indicators are displayed in bold, and the second-best indicators are presented in italics. ↑ indicates that a higher value represents better performance; ↓ indicates that a lower value represents better performance).

Model	MineDataset		LOL-V1		
	NIQE ↓	BRISQUE ↓	PSNR ↑	SSIM ↑	LPIPS ↓
EnlightenGAN	4.8638	26.2404	18.0146	*0.8060*	0.1699
IceNet	5.0727	28.4791	12.6562	0.6007	0.2014
SCI	5.4510	26.9646	13.9904	0.6753	0.1956
URetinex-Net	5.4446	30.2414	**20.2525**	**0.8788**	**0.0969**
Retinex-Net	5.5681	25.0122	*18.1257*	0.7867	0.1940
Zero-DCE	5.0139	29.5111	15.6161	0.7364	0.2174
Mine-DCE	*4.6012*	**18.9365**	17.3150	0.7694	*0.1584*
Mine-DCE-YDT	**4.5374**	*19.0268*	15.3885	0.7555	0.1589

**Table 4:** Comparison of detection performance. (The best indicators are displayed in bold in the table).

Model	Precision	Recall	mAP@0.5	mAP@0.5:0.95
Zero-DCE + YOLOv11n	0.969	0.900	0.948	0.795
Mine-DCE + YOLOv11n	0.977	0.932	0.962	0.839
Mine-DCE-YDT + YOLOv11n	**0.986**	**0.956**	**0.975**	**0.861**

**Table 5:** Comparison of computational complexity.

	GFLOPs (G)	Params (K)	Inference (ms)	FPS
Zero-DCE	5.19	79.42	1.59	628.93
Mine-DCE	5.63	84.25	3.25	307.69

## Data Availability

The original contributions presented in this study are included in this article. Further inquiries can be directed to the corresponding author.
